# Spatially-resolved quantification of proteins in triple negative breast cancers reveals differences in the immune microenvironment associated with prognosis

**DOI:** 10.1038/s41598-020-63539-x

**Published:** 2020-04-20

**Authors:** Rachel L. Stewart, Anna P. Matynia, Rachel E. Factor, Katherine E. Varley

**Affiliations:** 1 Department of Pathology and Laboratory Medicine, University of Kentucky College of Medicine, Lexington, KY USA; 2 Department of Pathology, ARUP Laboratories, University of Utah Medical Center, University of Utah, Salt Lake City, UT USA; 3 Department of Oncological Sciences, Huntsman Cancer Institute, University of Utah, Salt Lake City, UT USA

**Keywords:** Prognostic markers, Translational research, Breast cancer

## Abstract

Triple negative breast cancer (TNBC) is an aggressive breast cancer subtype. Recent studies have shown that MHC class II (MHCII) expression and tumor infiltrating lymphocytes are important prognostic factors in patients with TNBC, although the relative importance of lymphocyte subsets and associated protein expression is incompletely understood. NanoString Digital Spatial Profiling (DSP) allows for spatially resolved, highly multiplexed quantification of proteins in clinical samples. In this study, we sought to determine if DSP could be used to characterize expression of MHCII and other immune related proteins in tumor epithelial versus stromal compartments of patient-derived TNBCs (N = 10) using a panel of 39 markers. We confirmed that a subset of TNBCs have elevated expression of HLA-DR in tumor epithelial cells; HLA-DR expression was also significantly higher in the tumors of patients with long-term disease-free survival when compared to patients that relapsed. HLA-DR expression in the epithelial compartment was correlated with high expression of CD4 and ICOS in the stromal compartment of the same tumors. We also identified candidate protein biomarkers with significant differential expression between patients that relapsed versus those that did not. In conclusion, DSP is a powerful method that allows for quantification of proteins in the immune microenvironment of TNBCs.

## Introduction

Triple-negative breast cancer (TNBC) is an aggressive subtype of breast cancer that is associated with high rates of local recurrence and a propensity for distant metastases^1^. Clinically, TNBC is defined by the absence of three biomarkers that are used to inform treatment decisions: ER (estrogen receptor), PR (progesterone receptor), and HER2. While traditional predictive biomarkers are not expressed in TNBCs, there has been considerable interest in quantifying the host anti-tumor immune response in patients with TNBC to determine risk of recurrence and predict response to immunotherapy. Using RNA-seq of patient-derived TNBCs, we have shown that elevated expression of genes in the major histocompatibility complex Class II antigen presentation pathway (MHCII) is a favorable prognostic factor that is associated with long-term DFS^2^. This genomics discovery led us to develop and validate a clinical prognostic assay that measures expression of MHCII and tumor-infiltrating lymphocyte (TIL) genes in formalin-fixed, paraffin embedded (FFPE) clinical samples using NanoString technology^3^. We recently confirmed the prognostic significance of this MHCII Immune Activation assay in two independent cohorts of patients with TNBC^3^. Mechanistic studies support our clinical findings, as ectopic expression of MHCII has been shown to induce Th1-mediated anti-tumor immunity in mouse models of human cancer^4–6^.

We have shown that expression of MHCII mRNA in clinical samples is correlated with the presence of tumor-infiltrating lymphocytes (TILs) and with elevated expression of TIL genes including *CD3D*, *CD4*, and *CD8A*^3^. Our RNA-seq and NanoString gene expression studies were both performed using macrodissection of clinical TNBC specimens. While macrodissection enriches for carcinoma cells, both tumor and stromal infiltrating immune cells may be present in the macrodissected sample. Therefore, gene expression measured in clinical specimens is often reflective of both carcinoma cells as well as immune and stromal cells within the tumor microenvironment (TME)^7,8^. A logical question that arises is whether MHCII gene expression in patient tumors is simply a reflection of immune cells within the TME, or whether MHCII is expressed within tumor epithelial cells.

Protein expression and localization in clinical FFPE samples can be assessed using immunohistochemistry (IHC) or immunofluorescence (IF); however, the capacity for multiplexing is generally limited due to spectral overlap^9^. Furthermore, quantification of IHC is often performed using a semi-quantitative scale that can be subject to interobserver variability, particularly in the research setting^10^. Laser capture microdissection (LCM) is one method that can be used to isolate cell types and quantify protein and gene expression in distinct populations of cells, though it is technically challenging and difficult to scale^8^. NanoString Digital Spatial Profiling (DSP) is a highly multiplexed method that allows for protein quantification in spatially resolved, morphologically distinct areas within a single FFPE tissue section^9,11–15^. This method utilizes UV-photocleavable oligonucleotides that are conjugated to primary antibodies^13–15^. After incubation with a multiplexed cocktail of primary antibodies, oligonucleotides are cleaved in regions of interest (ROIs) using UV LED light and are then hybridized with NanoString optical barcodes for *ex situ* counting on an nCounter Analysis system^13–15^. In this study, we sought to determine if DSP can be used to characterize expression of MHCII and other immune related proteins in tumor epithelial versus stromal compartments of patient-derived TNBC samples.

## Results

**Figure 1:**
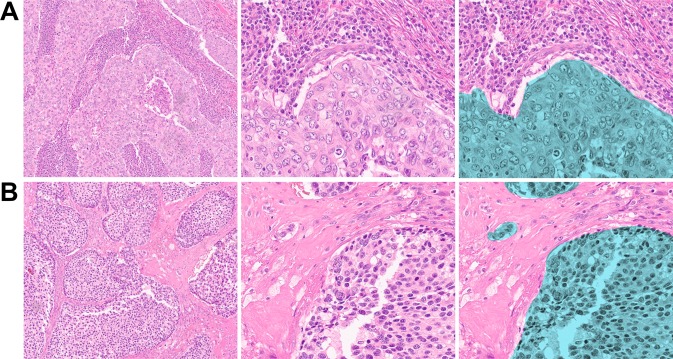
Stromal lymphocyte density in TNBCs. (**A**) Example of lymphocyte-rich TNBC containing sheets of syncytial epithelial cells and broad bands of stromal lymphocytes. (**B**) Lymphocyte-poor TNBC with hyalinized collagenous stroma and rare stromal-infiltrating lymphocytes. Right panel highlights epithelial compartments in green. Magnification: 10x(left-most panels); 20x(middle and right panels).

**Figure 2:**
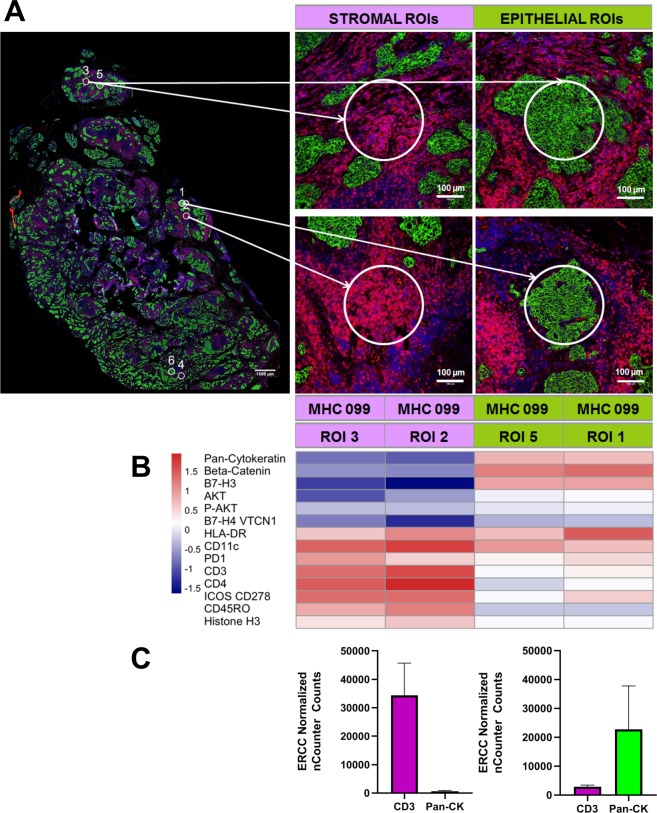
Region of interest selection and protein quantification in TNBC tumors. (**A**) Multi-label immunofluorescence was used to visualize tissue morphology and select regions of interest (ROIs). We selected six ROIs from each tumor, including three epithelial and three stromal ROIs. Four representative ROIs are shown from a single tumor. Magnification: 20x for all panels. (**B**) Epithelial and stromal ROIs had distinct patterns of protein expression that were consistent with the predicted cell types in each region. (**C**) CD3 expression was elevated in stromal ROIs whereas Pan-cytokeratin expression was elevated in epithelial ROIs.

TNBC tumor specimens have variable numbers of immune cells within epithelial and stromal compartments (Fig. 1). Additionally, stromal compartments within individual tumors can vary from lymphocyte-rich (Fig. 1A) to lymphocyte-poor (Fig. 1B). Stromal TIL density positively correlates with improved prognosis in patients with TNBC, although the relative importance of lymphocyte subsets and associated protein expression is incompletely understood^16^. The first main goal of this study was to determine whether DSP could be used to quantify proteins in morphologically distinct compartments within patient-derived TNBCs (*N* = 10). We selected distinct tumor cell epithelial and stromal ROIs and performed protein expression profiling using DSP; three epithelial and three stromal ROIs were selected from each tumor (Fig. 2A). We found that epithelial and stromal ROIs had distinct patterns of protein expression that were consistent with the predicted cell types in each region (Fig. 2B). Pan-cytokeratin expression was higher in epithelial ROIs, whereas the expression of CD3 and other immune cell proteins were significantly higher in stromal ROIs (Fig. 2B,C).

**Figure 3:**
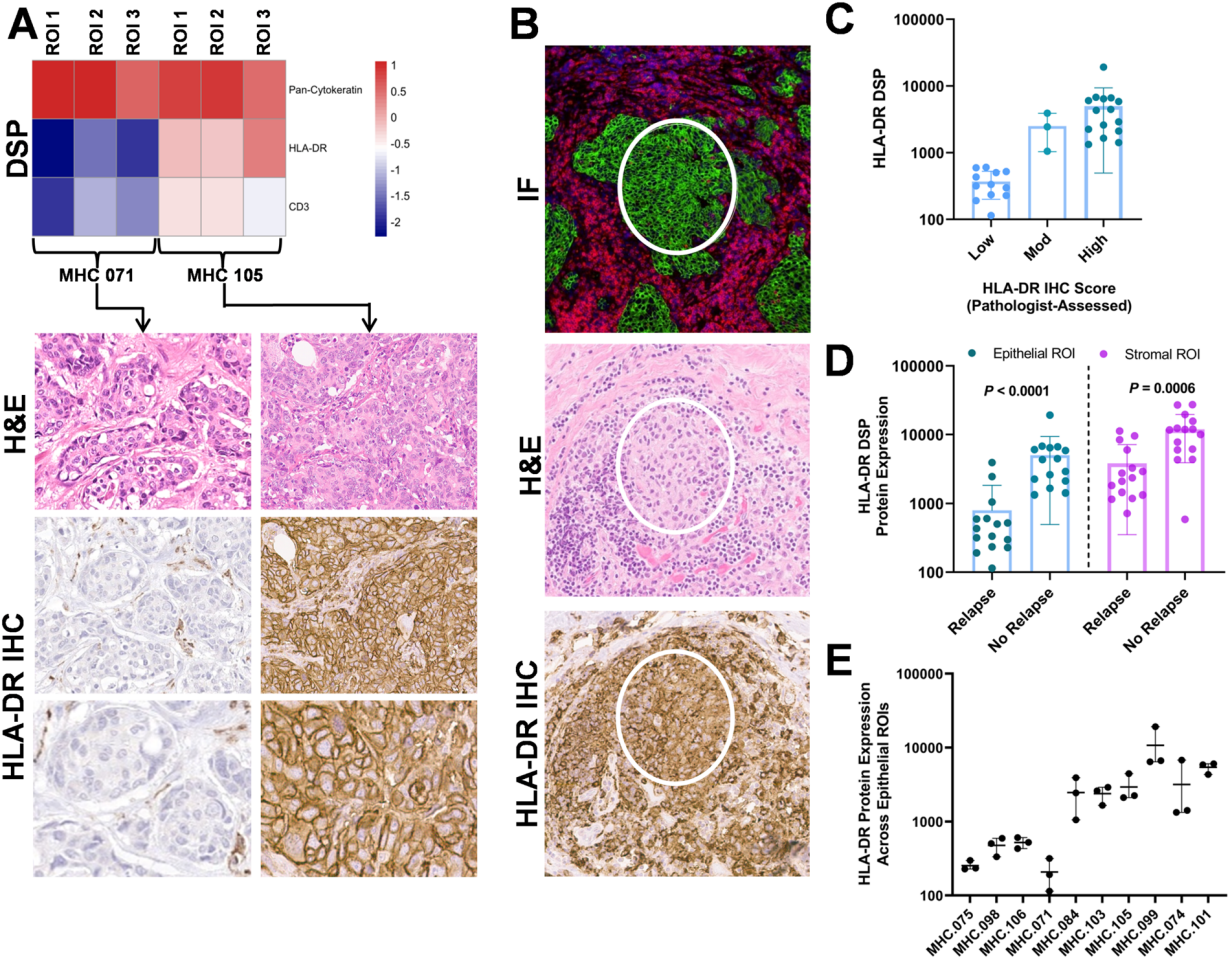
Comparison of HLA-DR expression in epithelial ROIs using DSP and IHC. (**A**) Examples of DSP measurements along with IHC staining are shown for a case with negative HLA-DR staining (Left panels) and for a case with elevated HLA-DR expression (Right panels). (**B**) Histomorphologic features were consistent between methods. (**C**) HLA-DR DSP counts and immunohistochemical scoring were positively correlated (Spearman *R*^2^ = 0.71). (**D**) HLA-DR protein expression in both epithelial and stromal ROIs was significantly higher in patients that did not relapse (P < 0.001; P = 0.006; Mann-Whitney tests). (**E**) HLA-DR DSP counts were relatively homogenous across the 3 epithelial ROIs from each tumor. Magnification for all images is 20x.

Traditionally, expression of MHCII was thought to be limited to antigen presenting cells (APCs) such as dendritic cells, B cells, and macrophages, although previous studies demonstrate that a subset of patient-derived TNBCs and TNBC cell lines can also express genes in the MHCII pathway^2^. To confirm that breast cancer epithelial cells can express MHCII proteins, we used DSP to evaluate HLA-DR expression in epithelial ROIs. We detected elevated HLA-DR protein expression in 18 TNBC epithelial ROIs from 6 tumors and low HLA-DR protein expression in 12 epithelial ROIs from 4 tumors (Fig. 3A). Pancytokeratin expression was likewise elevated within these HLA-DR positive ROIs, thus confirming epithelial origin of the signal. We then evaluated HLA-DR expression using IHC in these tumors, and results were concordant with DSP (Fig. 3A). Importantly, histomorphologic features were consistent between ROIs that had been assessed using DSP, H&E, and IHC (Fig. 3B). We then compared semi-quantitative IHC scores to normalized DSP protein expression counts, where we found that the measurements were highly correlated between the two methods (Spearman correlation coefficient *R*^2^ = 0.71; Fig. 3C). Additionally, HLA-DR protein expression was relatively homogenous across the 3 epithelial ROIs from each tumor (Fig. 3E). These findings validate the DSP detection of HLA-DR expression in epithelial tumor ROIs and confirm that a subset of patient-derived breast cancer epithelial cells can express MHCII proteins such as HLA-DR, which is consistent with our previous observations^2,3^, as well as with those of other investigators ^4,17–21^.

**Figure 4:**
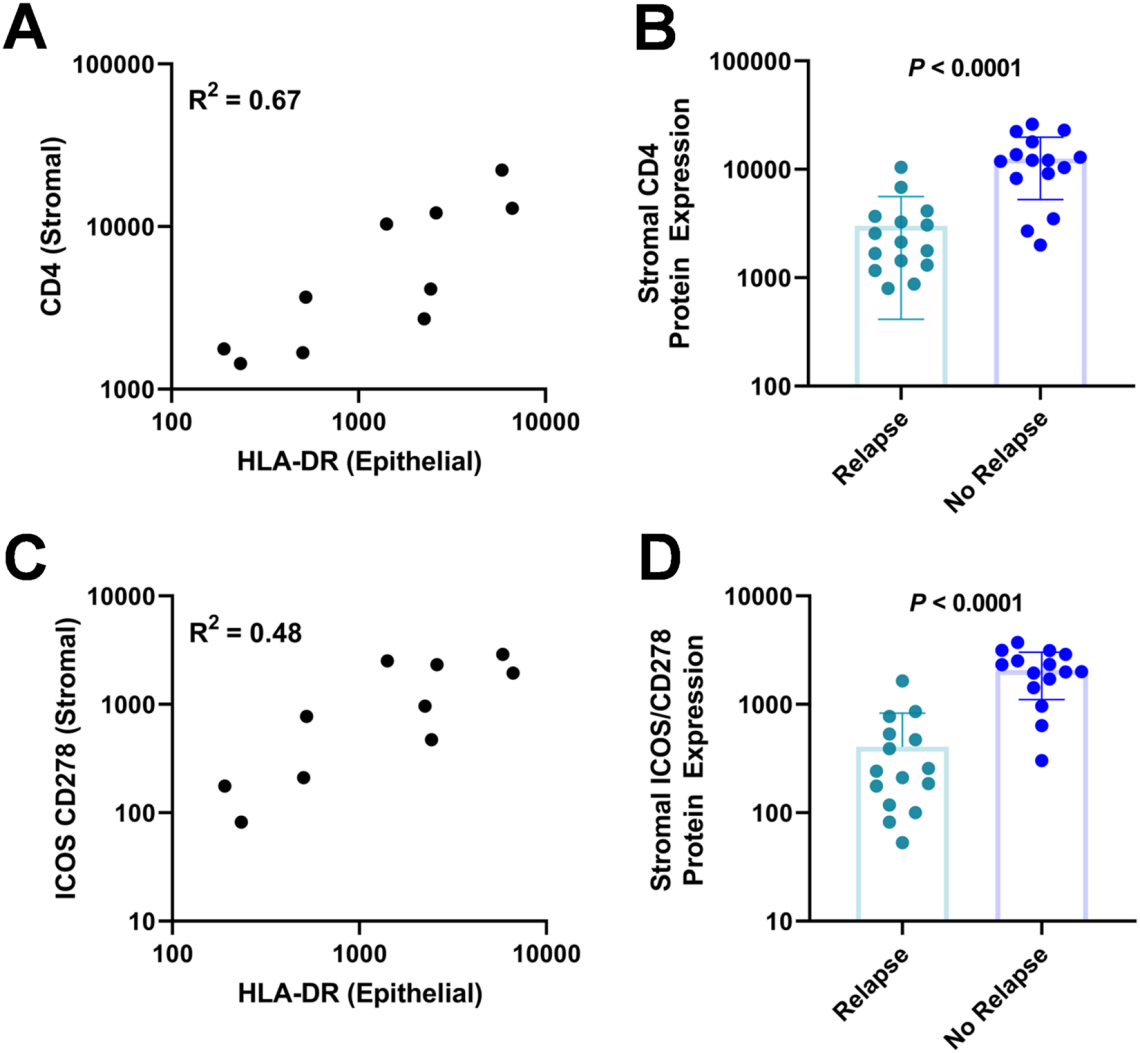
Epithelial HLA-DR expression is correlated with stromal CD4 and ICOS expression. (**A**) By linear regression, epithelial HLA-DR expression was positively correlated with expression of CD4 in the stroma (P = 0.0040; Pearson R^2^ = 0.67). (**B**) Stromal CD4 expression was significantly higher in patients with long-term DFS (P < 0.0001). (**C**) Epithelial HLA-DR expression was positively correlated with expression of ICOS (CD278) in the stroma (P = 0.0273; Pearson R^2^ = 0.48). (**D**) Stromal ICOS (CD278) expression was significantly higher in patients with long-term DFS (P < 0.0001).

We have previously demonstrated that elevated expression of *HLA-DRB1* mRNA in patient-derived TNBC tumors is significantly associated with long-term disease-free survival (DFS)^3^, though it has been unclear whether stromal or epithelial expression is more predictive of patient outcomes. Using DSP, we found that HLA-DR protein expression in both epithelial and stromal ROIs was significantly higher in patients with long-term DFS when compared to patients that relapsed (*P* < 0.001; Fig. 3D). Notably, the magnitude of differential HLA-DR expression between patient groups was more pronounced in the epithelial compartment (Fig. 3D). This finding is consistent with the hypothesis that aberrant expression of MHCII expression by TNBC epithelial cells results in the presentation of tumor-specific neoantigens to CD4+ T cells, thus contributing to the host anti-tumor immune response and improving patient outcomes^2^. In agreement with this hypothesis, we found that epithelial expression of HLA-DR was highly correlated with stromal expression of CD4 ((Pearson correlation coefficient *R*^2^ = 0.67; Fig. 4A), likely representing recruitment of CD4+ T lymphocytes to the tumor microenvironment. Correspondingly, CD4 protein expression in stromal ROIs was significantly higher in patients with long-term DFS when compared to patients that relapsed (P < 0.0001; Fig. 4B). In addition, we found that epithelial HLA-DR expression was highly correlated with stromal expression of ICOS (CD248; Pearson correlation coefficient *R*^*2*^ = 0.48; Fig. 4C), and that ICOS expression is significantly higher in patients that did not experience relapse (P = 0.0001; Fig. 4D). ICOS is a T-cell co-stimulator that enhances T-cell responses including proliferation and lymphokine proliferation; thus, it may mediate host anti-tumor immunity.

**Figure 5:**
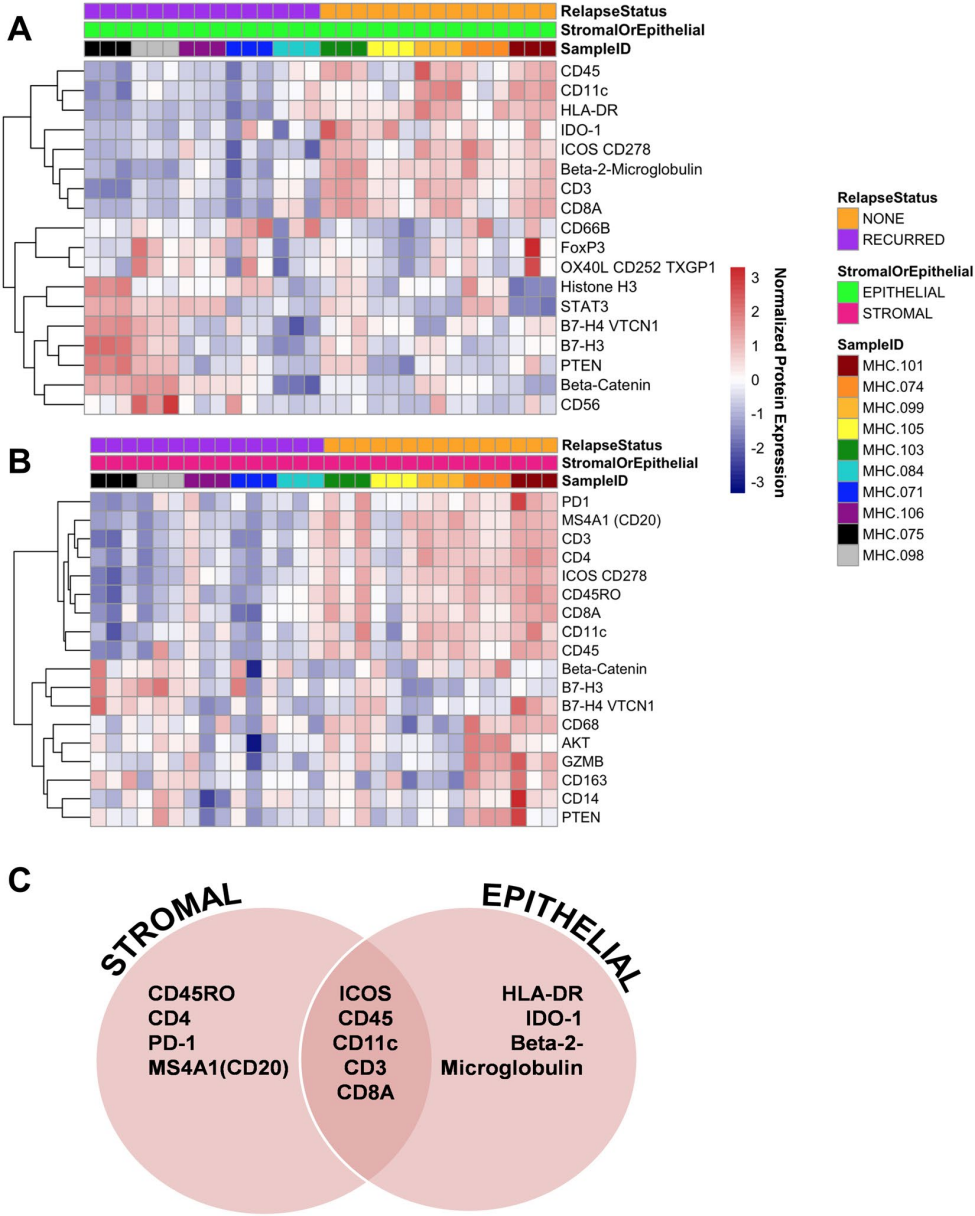
Differential protein expression and association with relapse status. DESEQ2 was used to identify proteins with significant differential expression between patients that relapsed versus those with that did not (adjusted p-values < 0.05). Heatmaps depict the quantification of proteins that were identified as significant in epithelial (**A**) and stromal (**B**) ROIs. (**C**) Overview of significantly elevated proteins in patients with long-term DFS. A subset of these proteins was found to be significantly elevated across both epithelial and stromal ROIs.

The multiplexed protein quantification provided by DSP allowed us to identify other proteins, besides HLA-DR, that may be involved in host immune response and that that may ultimately influence patient outcome. In order to identify proteins that were differentially expressed between tumors with disparate clinical behavior, we analyzed DSP data using DESEQ2. We observed striking differences in protein expression between tumors from patients that relapsed versus those that did not, and notably, we discovered that there were differences between the significant proteins within epithelial versus stromal ROIs (Fig. 5A,B). Among patients that did not relapse, we found that elevated expression of HLA-DR, IDO-1, and Beta-2 microglobulin were significant only within the epithelial compartment, whereas CD45RO, CD4, PD-1, and MS4A1 were significantly elevated in only the stromal compartment (Fig. 5A–C; Supplementary Table 1). We also identified 5 proteins that were significant across both epithelial and stromal ROIs (ICOS, CD45, CD11c, CD3, and CD8A; Fig. 5C). Future studies are warranted to determine if these candidate prognostic protein markers are significant in larger cohorts, and to investigate their role in anti-tumor immunity.

## Discussion

NanoString DSP is a unique method that allows for multiplexed protein quantification in clinical patient samples^11–15^. The method is gaining traction in the immune oncology environment and has been used to develop signatures that are predictive of patient response to immune checkpoint inhibitors^22,23^. We applied DSP to patient-derived TNBC tumors in order to interrogate differential protein expression in tumor epithelial and stromal compartments between patients who relapsed and those who did not. DSP provided quantitative protein expression measurements that allowed us to gain new insight into the complexities of the breast cancer immune microenvironment. The ability of DSP to simultaneously quantify multiple protein biomarkers in clinical samples is promising; however, extensive validation studies are required before this technology can be applied to guide patient care.

We applied DSP to patient-derived TNBCs, where we confirmed that a subset of breast tumors has elevated expression of HLA-DR in epithelial tumor cells. We also established that HLA-DR protein expression is significantly higher in the tumors of patients with long-term DFS compared to patients who relapsed. This finding was significant in both epithelial and stromal ROIs, although HLA-DR protein expression in the epithelial compartment of breast tumors was a better discriminator of patient outcome than it was in the stromal compartment. We also found that HLA-DR expression in the epithelial compartment is correlated with high expression of CD4 and ICOS in the stromal compartment of the same tumors. This finding is consistent with our hypothesis that MHCII positive TNBC epithelial cells present antigen and activate CD4+ T cells^2^. Although antigen presentation is typically thought to be limited to APCs, there is evidence in the literature to suggest that unique epithelial subsets may be capable of antigen presentation. For example, MHCII+ thyroid follicular epithelial cells have been shown to present viral peptide antigens to human T cells^24^. More recently, corneal epithelial cells were shown to induce MHCII upregulation in response to HSV-1 infection; these epithelial cells were found to have amateur antigen-presenting capabilities and could induce CD4+ T cell proliferation *in vitro*^25^. Our findings suggest that MHCII expression in epithelial TNBC tumor cells is an important factor in host immune response.

In addition to confirming expression of HLA-DR in tumor epithelial cells, the multiplexed protein quantification provided by DSP allowed us to identify novel candidate prognostic biomarkers that could enhance the power of existing MHCII biomarker assays, and inform future functional studies of the anti-tumor immune response. We found that tumors with elevated expression of HLA-DR expression in epithelial ROIs had elevated expression of CD4, ICOS, CD45RO, PD-1, CD11c, CD8 and MSA4A1 in the stroma. This result suggests that, in addition to CD4+ T helper cells, multiple types of immune cells may be present in the tumor microenvironment including memory T cells (CD45RO), dendritic cells (CD11c), cytotoxic T-cells (CD8A), and B cells (MSA4A1/CD20). In summary, DSP provided a novel and powerful method examining the immune microenvironment of TNBCs and identifying proteins associated with prognosis.

## Methods

### Patient-derived tissue sections

This project was performed under an approved University of Utah IRB protocol (#24487). Since the tissue specimens were leftover, de-identified archival FFPE blocks, the IRB waived the requirement for informed consent. Patient-derived TNBC specimens were collected as previously described^3^. All specimens were de-identified by the Huntsman Cancer Institute Biorepository and Molecular Pathology Shared Resource facility. For this study, a subset of tumors (*N* = 10) with adequate pathologic material in the archives were selected for protein expression profiling using NanoString DSP. In order to interrogate the immune microenvironment of TNBCs with disparate clinical behavior, we selected samples from patients that experienced disease recurrence (*N* = 5) and samples from patients that remained disease-free (*N* = 5) after a median follow-up time of 7.4 years. Clinical characteristics of these patients are provided in Supplementary Table 2. Only primary stage I-III breast cancers were included in the study. Detailed clinicopathologic and outcome data were obtained through review of the medical record.

### NanoString DSP and gene expression analysis

The DSP assay was performed on FFPE TNBC tumor sections through the NanoString Technology Access Program service. The specimens were sent to NanoString and ROI selection was performed through a web-based interface by a study team pathologist with the assistance of NanoString scientists. The DSP workflow can be divided into four main steps: (1) Tissue staining and imaging (2) Region of interest selection; (3) Cleavage and aspiration of oligonucleotide tags; (4) Hybridization and counting using the NanoString nCounter system. For this study, tissue sections (4 µm) were stained using a multiplexed cocktail of primary antibodies; each antibody was conjugated to a unique, UV-photocleavable indexing oligonucleotide tag. For this experiment we included all DSP validated antibodies-oligo conjugates available at the time of the study. A complete list of protein targets is provided in Supplementary Table 3. Tissue sections were imaged using three-color immunofluorescence in order to guide ROI selection. Morphology markers included CD3, pan-cytokeratin, and DAPI. A board-certified pathologist selected three epithelial ROIs and three stromal ROIs from each TNBC tumor section; each ROI measured 300 µm. UV LED light was used to release oligonucleotide tags from discrete ROIs. These tags were then aspirated, transferred to a 96-well plate and were then hybridized to target-specific barcode-labeled probes, as has been previously described^26^. Using the nCounter Prep Station, hybridized probes were immobilized in a cartridge, excess probe was washed, and the cartridge was transferred to the nCounter Digital Analyzer for analysis. All DSP values for the 39 markers are included in Supplementary Table 4.

### Immunohistochemistry (IHC)

IHC staining for HLA-DR was performed as previously described^3^. Briefly, 4 µm FFPE sections were stained using an HLA-DR antibody (Santa Cruz Biotechnology (sc-53319) in a Ventana BenchMark™ ULTRA autostainer. Sections were de-paraffinized and subject to antigen retrieval using Cell Conditioning 1 (CC1, pH 8.5) for 64 minutes at 95 °C. The primary antibody was applied at a concentration of 1:1000. Interpretation was performed as previously described^3^.

### Statistical analysis

Statistical analyses were performed using GraphPad version 7.0 C and R version 3.5.0. Raw counts for each of the photo-cleavage fluorescent probes corresponding to each of the proteins measured in the DSP assay were normalized to engineered RNA sequences (ERCC RNA controls) to correct for background signal in the assay. DESEQ2 version 1.20.0^27^ was used to identify proteins with significantly different normalized counts (adjusted p-value < 0.05) between stromal and epithelial ROIs as well as tumor from patients who recurred and those who did not. Heatmaps of log normalized protein counts were created using the R package ‘pheatmap’ version 1.0.10. Differences between groups were analyzed using Fisher exact test, 2-tailed t test with Welch correction, or 1-way analysis of variance with post hoc Tukey test.

## Supplementary information

Dataset 1.

Dataset 2.

Dataset 3.

Dataset 4.
